# Conveyor belt restraining in natural hazardous wind events

**DOI:** 10.1038/s41598-025-06135-1

**Published:** 2025-10-27

**Authors:** V. Golovanevskiy, A. Kondratiev

**Affiliations:** 1 Curtin University, Kent Street, Bentley, Perth, WA 6008 Australia; 2 O.M. Beketov National University of Urban Economy in Kharkiv, 17 Marshal Bazhanov St., Kharkiv, 61002 Ukraine; 3 University of Cambridge, Trumpington Str., Cambridge, CB2 1PZ UK

**Keywords:** Natural hazardous wind events, Conveyor belt, Irregular aerodynamic loading, Structural integrity, Optimal restraining interval, Finite element analysis, Divergence, Flutter, Mechanical engineering, Aerospace engineering, Civil engineering, Natural hazards

## Abstract

In preparation for the natural hazardous wind events such as tornados and tropical cyclones, open-air conveyor belts used by Australian mining industry for overland bulk materials transportation are physically restrained by tying them to their structural frames with rigid steel tie-downs over set regular intervals. This study examined a restrained section of a commercial type of conveyor belt focussing on discerning the most accurate approach for optimising the belt restraint interval value. Structural integrity of the belt was analysed with strength of materials, numerical modelling, and aerodynamic methods for a range of airflow angles and for airflow velocities of up to and including category 5 cyclonic events. It was found that strength of materials approach could be used as a rough approximation only, with somewhat fine-tuned results provided by the numerical methods and the conventional aerodynamic techniques. The most accurate results were obtained from introducing dynamic component of the conveyor belt’s elasticity into the aerodynamic flexural–torsional flutter treatment of the belt, with this approach delivering an over 30% higher minimal value of the length of the restrained section of the belt at design safety factor of 1.5. This larger restrained section length will lead to a corresponding reduction of the number of belt tie-downs and this, in turn, will translate into strong operational efficiencies while also delivering significant improvements of personnel safety. Additionally, the results of our work improve understanding of the behavior of open-air overland conveyor belts under the irregular aerodynamic loading conditions of natural hazardous wind events.

## Introduction

Australia is one of the world’s leading mining nations, producing and processing a wide range of hard commodities including metal ores and coal. The Pilbara in Western Australia and the Bowen Basin in Queensland are particularly significant Australian mining regions by both the scale of activity and the land area, covering almost 180,000 km^2^ in the northwest of Western Australia and over 60,000 km^2^ in central Queensland, respectively.

Both these regions are often subjected to extreme natural wind events such as tropical cyclones and tornados, with their frequency and severity increasing in the last 50 years^1,2^. It has been reported that 15% to 20% of the world’s total annual number of tropical cyclones develop over the Australian region, covering the northern part of Australia and neighbouring territories including Indonesia, Papua New Guinea, and Fiji^3^. On average five to six tropical cyclones per year made landfall in Australia between 1970 and 2005, mostly over northwest Western Australia and northeast Queensland. About half of them were categorised as severe, i.e., reaching maximum intensity of category 3 to 5 of the Australian Bureau of Meteorology classification, with maximum mean wind speed from 160km/h to over 200km/h and from 165km/h to greater than 279km/h respective wind gusts^4–6^. Often accompanying tropical cyclones heavy rainfall causes flooding inundating low-laying coastal areas, disrupts transportation, causes soil erosion and thus exacerbates the overall destructive tropical cyclones impact on both civil and industrial infrastructure^6–9^. 152 tropical cyclones within the Australian Exclusive Economic Zone or over the Australian mainland have been categorised as severe between 1970 and 2015, with their majority concentrated in the Western Australia region^10^. These extreme natural wind events often cause damage to property and critical infrastructure. As an example, the average total annual cost of tropical cyclone impacts in Australia from 1967 to 1999 was $8.8 bln AUD, or about 25% of the total cost of all natural hazardous events including floods, earthquakes and forest fires^11^.

Most of the Australian mining and mineral processing operations are situated in remote locations, often with tens and hundreds of kilometers distances between mine sites, processing plants and essential infrastructure such as railways and ports. With Australia’s vastness and predominantly flat terrain, the country’s mining industry primarily relies on bulk materials transportation by open-air overland conveyors, where mined and processed commodities are transported over multi-kilometer distances by reinforced-rubber conveyor belts. Single-flight open-air conveyors from several hundreds to several thousands metres long are a common feature of the Australian mining industry landscape, with some conveyors up to tens of kilometres long. As conveyors are high-cost, key infrastructure assets, every measure is taken to protect them from potential damage in cyclonic conditions, including belt material tears and stretches. The cost of such damage to the conveyor then includes belt repair costs and lost production time losses of up to a 100 thousand dollars for every hour the conveyor is out of service, often amounting to several million dollars.

In preparation for cyclone arrival, conveyor belts are physically restrained with mechanical tie-downs by personnel crews over regular 12.2m intervals at fixed points along the structural conveyor frames, with this same restraint interval used with all cyclone categories. This cyclone preparation procedure has been in use since the 1970s and has worked well, with no belt damage due to wind loading documented in the last several decades. However, to the best of our knowledge no justification of the 12.2 m restraint interval is available, including public domain and proprietary literature and conveyor systems design codes alike. It appears plausible that this numerical value could be the result of conversion of units of length as part of Australian metrication in the 1970s and 1980s, when an integer value of 40 ft in the Imperial units would become a non-integer value of 12.2 m in the metric notation. Nevertheless, while this could potentially explain the 12.2 m value the engineering basis of the original i.e., 40 ft value of the restraint interval remains similarly lost in history.

Restraining and freeing-up of conveyor belts is both a high-cost and a hazardous task, often exacerbated by a significant, i.e., in many instances several kilometres long, conveyors lengths. Reducing the number of conveyor belt restraints by increasing the regular belt restraint interval would therefore translate into significant improvements of personnel safety while at the same time delivering strong operational efficiencies. However, with the justification of the current restraint interval unavailable, it is unknown what, if any, increase of this value can be tolerated without an unacceptable risk of belt’s damage from the cyclonic wind loads. The potential gains of a reduced number of belt restraints warrant examination of the engineering basis of design of the belt restraint interval, and this requires analysis of the aerodynamic behavior of conveyor belt in cyclonic conditions. However, to date such an analysis remains an unexplored area of scientific enquiry.

## Materials and methods

### Overland conveyor design considerations

**Fig. 1:**
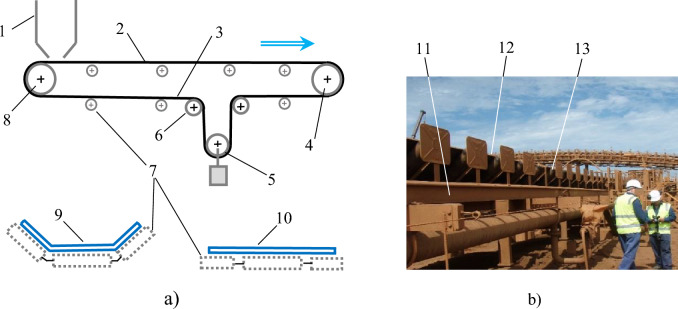
Open air overland bulk materials transportation conveyor. (**a**) conveyor schematics, (**b**) open-air overland conveyor – partial side view. 1 – bulk material feeder; 2, 3 – belt delivery and return sections, respectively; 4 – head pulley; 5, 6 – take-up and bend pulley, respectively; 7 – supporting rollers with end dust shields; 8 – tail pulley; 9 – troughed belt delivery section profile; 10 – flat (or quasi-flat) belt return section profile; 11 – structural conveyor framework; 12 – belt delivery section; 13 – supporting rollers with dust end-shields (Belt travel direction shown by arrow).

Schematic of an overland minerals transportation conveyor system and a view of a delivery section of a typical Pilbara open-air overland conveyor are shown in Fig. 1. (From here on, the terms conveyor belt, conveyor, belt, and belt conveyor denote the delivery, i.e., troughed, section of the conveyor belt, unless used in a specific context of a conveyor system assembly/infrastructure).

Referring to Fig. 1a), the head and tail pulleys physically restrain movement of the belt relative to its fixed structural subframe in the horizontal direction along its main (i.e., longitudinal) axis. Movement of the belt in the vertical downward direction is arrested by the supporting rollers, while the vertical upward movement of the delivery section of the belt is counteracted only by its weight and the restoring elastic forces. Figure 1b shows that the entire conveyor system, including the conveyor belt and structural framework with ancillaries, is fully exposed to ambient airflow.

**Fig. 2:**
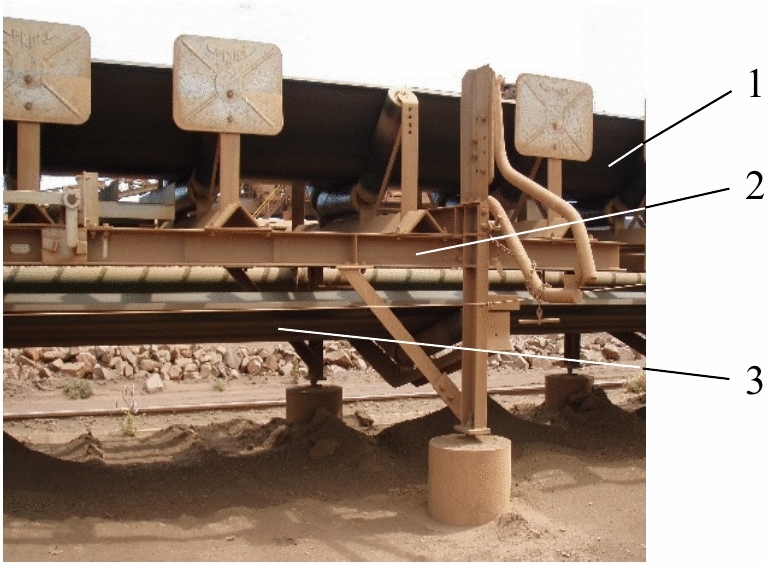
Belt delivery and return sections. 1 – underside of troughed delivery section; 2 – delivery section subframe; 3 – flat / slightly concaved return section.

As the belt is not physically attached to the conveyor structure and instead simply rests on the supporting rollers (Fig. 2), under the wind load it is potentially free to twist and/or move in the vertical-upward direction.

As seen in Fig. 2, the upward movement of the return section of the belt is restrained by the subframe of the belt delivery section in close proximity. Additionally, as the return section of the belt is nominally flat (i.e., only slightly concaved under its own weight) its transverse profile area is too small for the transverse wind load to cause the return section to move transversely. In contrast, vertical-upward movement of the delivery, i.e., troughed section of the belt is unimpeded, and its transverse profile area is sufficiently large for the wind load to cause it to move. As a result, under the wind load the delivery section of the belt can lift, twist, slide sideways, and sway.

Conveyor belts of many types, differing by physical-mechanical and dimensional properties depending on their designated service duty, are used for the overland bulk materials transportation. The work reported here was carried out for a commercial steel-cord reinforced rubber conveyor belt of one of the most common types, with its relevant parameters shown in Table 1.

**Table 1:** Conveyor belt parameters.

Parameter		Value
Width, nominal, mm		1, 800
Thickness, nominal, mm		30.0
Density, nominal 10^–3^ × kg/m^3^		1.515
Tensile strength, MPa	axial	199.0
	transverse	19.9
Tensile modulus, MPa	axial	4, 728
	transverse	15.6
Shear modulus, MPa	axial	12.0
	transverse	25.2
Poisson ratio, nominal		0.48

With the exception of Tensile and Shear moduli measured in our earlier work^12^, all parameters’ values were provided by the conveyor belt vendor.

### Conveyor belt restraining: current Australian best industry practice

When a cyclone warning is issued by the Australian Bureau of Meteorology, 24 hours prior to the cyclone threat the feeding of the conveyors with the bulk material is ceased. The conveyors are run until the bulk material they carry is fully unloaded, and the conveyors are then stopped. Next, the belts are restrained from potential movement under wind loads by physically securing (i.e., tying down) their delivery sections to the structural conveyor frames at fixed points over regular 12.2 m intervals. While a small number of short, i.e., up to several hundred metres long, conveyors have recently been fitted with automated, i.e., electrically operated (i.e., engaged and disengaged) belt restraints mostly on a trial basis, the cost of automating restraints on long conveyors is prohibitive. Consequently, the majority of the conveyors are fitted with mechanical i.e., manually operated restraints.

**Fig. 3:**
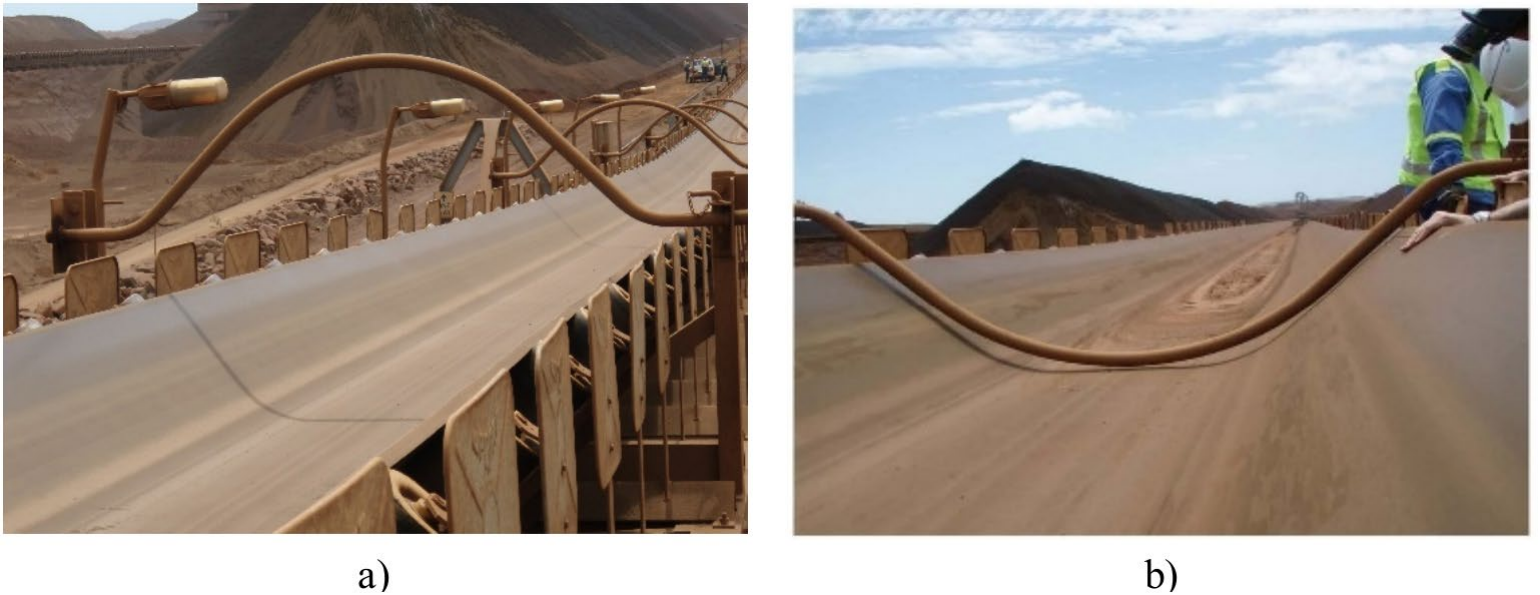
Open-air conveyor belt restraints. (**a**) disengaged position; (**b**) engaged position.

Belt restraining is carried out by personnel crews driving along the entire exposed length of the conveyor and physically securing the delivery section of the belt over the 12.2 m regular intervals with mechanical restraints held in position with chain tie-downs. After the cyclone, personnel crews carry out this operation in reverse i.e., driving along the conveyor and disengaging and removing the restraints to free-up the conveyor belt in preparation for its re-start. Several designs of belt restraints are currently in use, with an example of a single-piece rigid curved restraint shown in Fig. 3.

With some of the conveyors several kilometers long, several personnel crews are often dispatched to operate simultaneously to ensure that the conveyors are secured in time for the cyclone and then freed-up efficiently post-cyclone for re-start.

## Structural integrity examination

### Aerodynamic environment considerations

**Fig. 4:**
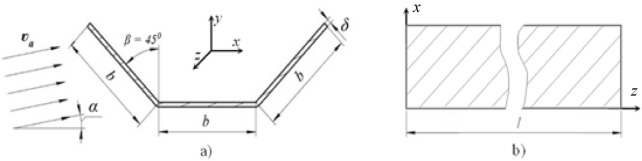
Conveyor belt restrained section schematics. (**a**) transverse cross-section; (**b**) side view.

The aerodynamic conditions created by tornadoes and cyclones are characterised by turbulent airflow, with its corresponding unpredictable effects on any object, including flow direction, affected area, and dynamic characteristics^2^. Recognising this, our work proceeds from the following assumptions (ref. Fig. 4).

(i) Regardless of the type of the aerodynamic flow and its specific area of action on the restrained section of the conveyor belt, line of action of the incident airflow (i.e., air gust) velocity vector, v_a_ , is given by its angle of attack, α (i.e., angle of airflow).
(ii) During the period of airflow interaction with the belt, the speed and the direction of the airflow are constant and hence the time factor can be disregarded.
(iii) The transverse cross-section of the belt over the entire length, l, of its restrained section has a rectilinear trough-shaped symmetrical profile consisting of three straight sections of equal length, b, and thickness, δ, with horizontal mid-section and two outer sections at β = 45^0^ angle to the mid-section.
(iv) The belt profile is subject to two aerodynamic forces i.e., lift force, F_y_, and drag force, F_x_, forces of weight, F_w_, and aerodynamic torque, M_z_. Forces F_y_ and F_x_ are applied at the centers of pressure of each linear section of the profile (at the points of application of the resultant aerodynamic forces), while forces F_w_ are applied at the centers of gravity of the sections (with the resultant force F_w_ applied at the centroid of the full cross-section).

Additionally, it is also assumed that the airflow is not distorted by the conveyor support rollers with their end dust-shields (ref. Fig. 1). Thus, at any point along the restrained section of the belt there is a laminar (unseparated) airflow around an idealized troughed conveyor belt profile.

Wind pressure is a significant aerodynamic factor of a turbulent airflow, since under its action, in general, the magnitude, direction and application point of the wind load rapidly change^13^. The effects of a turbulent airflow on engineering structures resulting from the dynamic character of the wind load and from wind gusts in particular, can be accounted for with a conditional increase in the estimated static load on the structure (i.e., the “Gust Loading Factor (GLF)” approach). This is achieved by multiplying the mean wind load value by a dynamic factor (i.e., GLF) that considers the impact of the fluctuating component of the wind load and wind pressure and the effects of gusts^14,15^. The GLF values are set by structural design codes and standards used by the countries’ regulatory authorities^16,17^.

Conveyor belt profile experiences aerodynamic forces and their rotational moments about pressure centres of the belt profile sections, and the mass forces (i.e., weights) of the sections. Under the dynamic wind loading conditions and with the above assumptions, a restrained section of the belt experiences internal reactive elastic forces that oppose these external, i.e., aerodynamic and mass, forces thereby bringing the restrained section of the belt into equilibrium. Assuming perfectly elastic material of the belt, several mathematical models can then be synthesized for the analysis of its aerodynamic behaviour. Strength and stiffness of the belt can be examined using the aerodynamic flow model introduced above and a structural model of the belt. In the first approximation, structural integrity of the belt can be analised with a conventional strength of materials (SM) approach. This can then be followed with numerical modeling (NM) using finite element methods, and finally with aerodynamic approaches (AA) taking into consideration aeroelasticity and flutter of the belt.

### Strength of materials approach

The conveyor belt in focus can be regarded as a structural element comprised of thin-walled components (sections) of low rigidity. Given the shape of the belt and the relationships between its characteristic dimensions, the most reasonable standard structural model for its representation in SM analysis is that of a thin-walled beam of open cross-section, where the beam is defined as a body whose length is many times its width and whose thickness is negligible^18^.

The main provisions of the theory of thin-walled beams were introduced by Timoshenko in 1936^19^, with its subsequent development by Vlasov in 1961^20^ and subsequently other researchers^21^ leading to the thin-walled beam models finding wide acceptance in the analysis of slender frames in various application including structural elements^22,23^, high-rise buildings^24,25^, wind turbine blades^26^, and aircraft structures^27,28^. Similar to any strength of materials (SM) approach, strength analysis using the thin-walled open cross-section beam model requires the adoption of several assumptions. As applicable to the work reported here, these assumptions include the non-deformability of the cross-section of the belt, linear dependence between the magnitude and line of application of aerodynamic force and the angle of airflow, and small transverse displacements of the belt’s longitudinal axis between the restraints (ref. Fig. 4).

Assuming uniformly distributed over the cross section of the belt wind load, its horizontal, q_z_, and vertical, q_y_, components (ref. Fig. 4b) can be found as

1 $$\left.\begin{array}{c}{q}_{y}=\left({C}_{y1}+{C}_{y2}+{C}_{y3}\right)\frac{{\rho }_{a}{v}_{a}^{2}}{2}b\\ {q}_{x}=\left({C}_{x1}+{C}_{x2}+{C}_{x3}\right)\frac{{\rho }_{a}{v}_{a}^{2}}{2}b\end{array}\right\}$$

where

C_yi_ and C_xi_ – coefficients of aerodynamic lift and drag of the restrained belt section, respectively; ρ_a_, v_a_ – density of air and air gust velocity; b – length of a single straight section of the cross-section of the belt.

Considering free torsion of the conveyor belt as a thin-walled open cross-section beam and accounting for the weight of the conveyor belt, bending and rotational moments acting on the belt and moment of inertia of its cross-section, and substituting in the values of longitudinal and transverse belt strength^9^, conventional SM transformations of Eq. (1) yield the following expressions for the critical (i.e., minimal) distance, l_cr_, between the belt restraints based on the maximum acceptable tensile, l_crσ_, and shear, l_crτ_, stress levels:

2 $${l}_{c{r}_{\sigma }}\le \sqrt{\frac{\sigma b\delta }{{K}_{d}\left[\left(0.132{C}_{y}+0.058{C}_{x}\right){\rho }_{a}{v}_{a}^{2}-0.75\rho g\delta \right]}}$$

3 $${l}_{c{r}_{\tau }}\le \frac{\tau b}{{K}_{d}\left(0.566{C}_{y}+0.53{C}_{x}\right){\rho }_{a}{v}_{a}^{2}}$$

where:

σ – tensile strength of the belt in the longitudinal direction; b – length of a single straight section of the cross-section of the belt; δ – belt thickness; K_d_ – dynamic loading ratio (K_d_ = 3); C_y_, C_x_ – coefficients of aerodynamic lift and drag; ρ_a_ , v_a_ – density of air and air gust velocity; ρ – conveyor belt material density; g – gravitational acceleration; τ –shear strength of the belt.

The analysis of the stress-strain state of the belt under its restrained torsion showed that, due to the low rigidity of the belt’s cross-section the values of the respective normal and shear stresses were significantly lower than bending stresses. For v_a_ =270 km/h ≡ 75 m/s (i.e., approaching the 77.5m/s upper limit of the category 4 cyclone gust velocity values range^6^), the restraint intervals determined with Eqs. (2), (3) were 33.8 m and 17.9 m from the tensile and shear strengths considerations, respectively. The very large, i.e., over 61% difference between these results is due to determining the mean shear strength value of the belt as the quadratic average of the axial, σ_l_, and the transverse, σ_t_, tensile strengths of the belt (τ = 0.6√(σ_l_ σ_t_)) and thus accounting for 10x difference between these tensile strength values of the belt in focus (ref. Table 1). Using the smaller of the two l_cr_ values, from the belt integrity considerations the minimum distance between the belt restraints calculated based on the SM approach was accepted as l_cr_ = 17.9 m.

### Numerical modeling

Wind loading effects on industrial and civil engineering structures are commonly analysed with physical experiment in wind tunnel^13,29,30^. However, while after full-scale site trials this approach remains the most reliable design tool of experimental aerodynamics^31,32^, ensuring full similarity of physical experiment and a real-life aerodynamic process is not always achievable. Additionally, physical constrains of the available equipment place restrictions on the configuration of objects that can be practically modelled in wind tunnel^33,34^. In the consideration of foils-like structures with their high dimensional ratios and of the conveyor belt in focus in our work in particular, with over 50 times the ratio between the width and thickness of the belt, wind tunnel experiments are unrealistic, if at all possible^35^. Instead, it is significantly more efficient to analyse aerodynamic characteristics of the belt and similar structures with NM using finite element (FE) analysis simulations software^31^ as, for example, was used for the analysis of a severe structural damage of an overland conveyor system, including both the framework and the conveyor belt, damaged in 1986 by a tornado in Iowa, USA^36^.

Under wind loading of extreme natural wind events, the restrained belt section undergoes deformations correspondent with the unpredictable wind behaviour. Due to the low stiffness of the belt, these deformations range from small to large, with the aerodynamic forces and pressure distribution profiles over the surface of the belt varying correspondently. It then follows that the numerical analysis of the belt should incorporate this dependence of the stiffness of the belt on the wind loading. However, modelling this non-linear and constantly changing wind loading / belt profile relationships presents a significant challenge as the functionality of the current FE software packages is limited to the use with the set (i.e., not changing under loading) models and with independence of the loading of the model on the model’s geometric shape.

The challenge of reconciling the airflow and the load-carrying capacity of the belt was addressed by its linearization, substituting the non-linearity with a sequence of three linear problems, each with set aerodynamics and geometric conditions, The airflow velocities used were 25 m/s in the first, 50 m/s in the second, and 75 m/s in the third linear problem. Computational Fluid Dynamics (CFD) and Computer-Aided Engineering (CAE) software packages were used in an iterative computational sequence. This involved determining the distribution of the aerodynamic forces over the surface of the belt using the CFD package, followed by the evaluation of the load-carrying capacity of the belt with the FE CAE modeling, including consideration of the mass forces of the belt. The results of the CAE calculations from the first computational sequence (i.e., for 25 m/s airflow) were then used as the input data for the next CFD / CAE step (i.e., for the 50m/s airflow), and this was then repeated for the 75 m/s airflow velocity.

For the modeling, airflow velocity v_a_ and angle of attack α were adopted as the initial parameters for the considered load cases, and the following assumptions were made:

- the oncoming airflow is laminar,
- the airflow flows around the isolated belt,
- the roughness of the belt is negligeable.

**Fig. 5:**
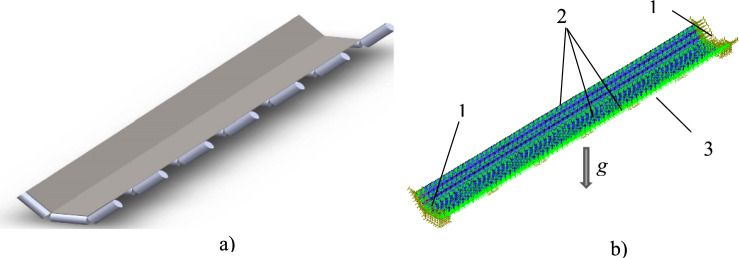
Restrained belt section: (**a**) 3D model (Restraints not shown); (**b**) FE model loading and boundary conditions; 1 – belt restraints; 2 – aerodynamic loading; 3 – supporting rollers.

Referring to Fig. 5, the FE model of the belt was loaded with positive pressures imported from the CFD calculations results and the loads due to the weight of the belt. The latter were accounted for by loading the FE model with **g** = 9.8 m/s^2^ gravitational acceleration. At the ends of the restrained section of the belt, restrictions were placed on all movements (i.e., fixed boundary conditions).

**Fig. 6:**
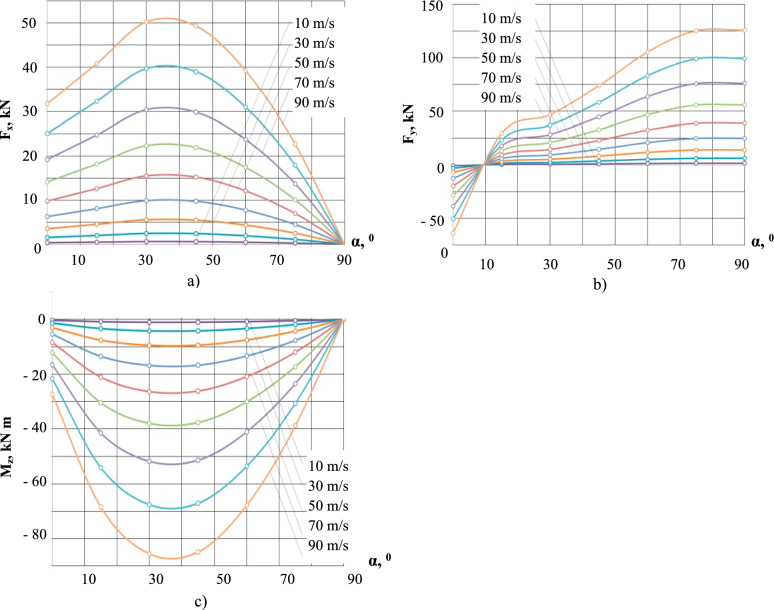
Aerodynamic forces and moments. (**a**) drag force; (**b**) lift force; (**c**) aerodynamic moment. (50 m/s – category 3 cyclone; 70 m/s – category 4 cyclone; 90 m/s – category 5 cyclone).

The distributions of the aerodynamic forces and moments for different v_a_ and α values obtained with numerical modeling (NM) are shown in Fig. 6. For all airflow velocities shown (i.e., from 10 m/s to 90 m/s in 20 m/s increments), the drag force and the aerodynamic moment reach their maximum values at *α* between 30^0^ and 40^0^, while the lift force attains its maximum at *α* between 75^0^ and 90^0^. The growth of the lift force with the increase of α is a consequence of the increase in the projection of the blown area of the belt onto the horizontal plane. At α < 9^0^, the lift force has a negative value – i.e., conveyor belt is pressed against the supporting rollers of the conveyor frame (Fig. 6b).

Calculations of the load carrying capacity of the belt included the use of a safety factor (SF), i.e., a multiplier of GLF, that evaluates stress in each stress node based on a chosen yield criterion taking into account the purpose of the facility, the specifics of its operation, operating conditions, and requirements that ensure its reliability and safety. For the work reported here, we adopted SF value of 1.5 as acceptable for structures the destruction of which does not endanger human life^37^.

**Fig. 7:**
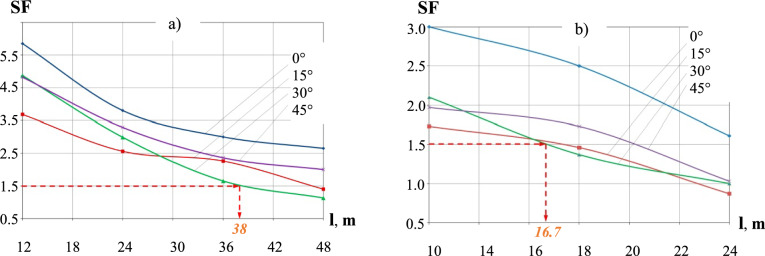
Restrained belt section lengths (Shown are the airflow angles). (**a**) v_a_ = 45 m/s; (**b**) v_a_ = 75 m/s.

Calculated restrained belt section lengths values for two airflow velocities and a range of airflow angles are illustrated in Fig. 7. As can be seen, increasing the air gusts velocity by over 66% i.e., from 45 to 75 m/s (i.e., the upper limit of category 2 and 4 cyclones, respectively)^6^ at SF=1.5 results in more than 50% reduction of the restrained section length from 38.0m to 16.7m, at *α* = 30^0^ for both airflow velocities.

As shown in Fig. 7b), for v_a_ = 75 m/s airflow at different angles of attack the shortest, and hence the most reliable to ensure no structural damage to the belt in the event of a cyclone, length of the restrained section of the belt is l_cr_ = 16.7 m, at safety factor SF = 1.5. We note that this l_cr_ =16.7 m value is lower than the l_cr_ = 17.9 m value obtained with the SM approach and propose that this is due to incorporating the wind loading / belt profile relationships in the iterative NM CFD / CAE computations, resulting in a more accurate analysis of the structural integrity of the belt as compared with the SM approach with its more rigid assumptions.

Referring to Fig. 7, increasing SF value from 1.5 to 2.0 reduces the minimal safe restraining interval value, l, from 16.7 m to approximately 10 m, while reducing SF from 1.5 to 1.2 extends the interval l to approximately 21 m. The “SF versus l” relationship can be further readily interrogated graphically for other SF values and for a range of angles of attack.

As demonstrated by the analysis in Fig. 7, the angle of attack plays a decisive role in determining the aerodynamic response of the belt. Specifically, the airflow direction corresponding to an angle of attack between 30° and 40° was found to induce the highest combination of aerodynamic forces and moments on the restrained belt section (ref. Fig. 6). This angular range consistently resulted in the most critical loading scenarios across all tested wind speeds. These findings indicate that belt orientation relative to prevailing wind direction is a key factor in the onset of instability.

### Aerodynamic approach

Aerodynamic considerations often play a critical role in the design and operation of structures subjected to wind loading such as civil engineering installations, surface vehicles, and aircraft. In aircraft design, structural components requiring aerodynamic input are ailerons, tails, rudders, and wings, all regarded as two-dimensional as their thickness is negligible compared with their width and length. The geometry of an aircraft wing in particular – with it’s thickness negligeable and its length many times its width – is similar to the conveyor belt geometry, and on the grounds of this similarity we propose that the restrained section of the belt be regarded analogues to an aircraft wing. Accepting this then leads us to further propose that the aerodynamic approaches developed for the treatment of the aircraft wing are also applicable to the restrained section of the belt. On this premise, the respective applicabilities of the aerodynamic treatments of the aircraft wing and the belt then differ only in their boundary conditions. I.e., while the wing is rigidly fixed at one end to the fuselage of the aircraft, the restrained conveyor belt section is rigidly fixed at both ends to the conveyor frame (i.e., by mechanical restraints).

**Fig. 8:**
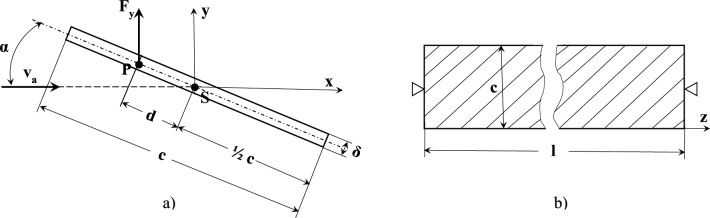
Belt cross section: aerodynamic model schematic. (**a**) cross-section; (**b**) side view. *F*_*y*_ aerodynamic lift force, *c, δ* cross-section length and thickness, *d* aerodynamic moment arm, *P* pressure centre, *S* shear centre, *v*_*a*_ airflow velocity; *α* airflow angle. (The largest d possible i.e., when F_y_ is acting normal to the cross-section plane is d = ¼ c).

With the troughed profile of the restrained section of the belt unfolded and flattened and the section therefore straightened into a plane, the schematic of the cross-section of the restrained section of the belt for its aerodynamic treatment is shown in Fig. 8.

The aeroelasticity phenomena normally considered as part of aerodynamic studies are distinguished as static or dynamic. Static aeroelasticity accounts for the reaction of the component/structure to the interaction of only aerodynamic and reactive (i.e., elastic) forces and is described by the mathematical model of divergence. In addition to these forces, dynamic aeroelasticity also encompasses inertia forces and is described by the mathematical model of flutter^38^. With reference to Fig. 8, determination of the critical airflow speed values of the aeroelastic phenomena is then carried out as follows.

#### Divergence

In aircraft aerodynamics, the most common divergence problem is the divergence of the torsion of the aircraft wing [385]. Here, the lifting forces twist the wing thereby increasing its local angle of attack which further increases the twist of the wing. This increased twist, in turn, further increases the angle of attack which, again, adds to the wing’s twist etc. This induced aerodynamic twist is opposed by the restoring i.e., elastic torque, with the maximum equilibrium reached at the so-called critical divergence speed of the aircraft determined by the maximum magnitude of the restoring elastic forces. If this divergence speed is exceeded, the aerodynamic twist of the wing will exceed the restoring elastic torque causing the wing to break. Applying this analogy to the conveyor belt torsion-divergence treatment, the aerodynamic forces deform the restrained section of the belt in such a way that the aerodynamic loads on the belt increase further. This increased loading then causes the belt to deform even more which further increases the aerodynamic loading and exacerbates the belt’s twist and so on. When this compounded aerodynamic loading on the belt becomes so large that the opposing elastic forces of the belt can no longer restore equilibrium, structural failure of the belt results.

The aerodynamic lift force, F_y_, acting normally to the airflow velocity vector v_a_ is found as

4 $${F}_{y}={C}_{y}\frac{{\rho }_{a}{v}_{a}^{2}}{2}A$$

where:

C_y_ – aerodynamic lift force coefficient (determined in virtual wind tunnel experiments for a given airflow angle); ρ , v_a_ – density of air and airflow velocity; A = c×l – characteristic aerodynamic area of the restrained section of the belt.

This lift force is applied at the centre of pressure of the profile in front of the centre of stiffness relative to which the section of the belt profile is twisted under the action of the aerodynamic moment M_a_ = F_y_×d, where d is the moment arm. This twisting is impeded by the restoring torsional moment of the elastic forces relative to the stiffness center of the profile, M_el_

5 $${M}_{el}=GJ\frac{{d}^{2}\varphi }{d{z}^{2}}$$

where:

G – torsion modulus (i.e. transverse shear modulus, ref. Table 1) of the material of the conveyor belt^12^, J – moment of inertia in torsion of the cross-section of the belt at the $\frac{{d^{2} \varphi }}{{dz^{2} }}$ relative twist angle.

Analogous to the model of torsion divergence of a straight wing of an aircraft^38,39^, the equilibrium condition for the single straight section of the cross-section of the belt is

6 $${M}_{a}+{M}_{el}=0$$

Solving this equation derives the critical divergence speed (i.e., the airflow speed) range from 6 km/h to 11 km/h.

However, in their normal service duty the overland conveyor belts are operated in winds severalfold stronger than these airflow values, with the upper airflow speed limit determined by the need to control the blowout of the bulk material carried by the belt rather than by the belt’s structural integrity considerations. On these grounds, as the critical airflow values of 8.5 ± 2.5 km/h are decidedly unrealistic the use of static aeroelasticity (i.e., divergence) approach for the analysis of structural integrity of the belt in cyclonic events is considered unsuitable.

#### Flutter

It is well known that the destruction of civil engineering structures such as bridges in high-speed airflow has a significant dynamic component and occurs as a result of their oscillations where the wind forces and structural motion are strongly linked^40^. As an example, this interactive forces-motion phenomena was shown to lead to the self-excitation of the Tacoma Narrows bridge as a result of its critical tortional oscillations, ultimately causing the bridge to collapse^41^. Similarly for a conveyor belt, when accounting for the dependence of its stiffness on the wind loading (ref. Sect. “Numerical modeling”), consideration of the behaviour of the belt under the dynamic wind loading conditions shows that, as the wind load oscillates the belt must oscillate in sympathy. It then appears reasonable to propose that the aerodynamic analysis of a restrained section of conveyor belt should include the input of the inertial forces and therefore be dynamic. More specifically, the aeroelastic approach should include the effects of the oscillations of the system and this leads to the consideration of the model of the flexural-torsional flutter as used extensively in civil and structural design and in aircraft engineering. Some of the recent relevant uses of this approach include investigations of flutter of pedestrian bridge and the Great Belt Bridge (Denmark) and modeling flutter of rectangular profiles^42–44^, aeroelastic analysis of a flexible aircraft wing in a coupled bending-torsion motion with the incorporated effects of wind gusts^45,46^. Also considered was the frequency of an aircraft wing subjected to flutter instability^47^, while experimental study of a model of a wing found that flutter takes place by the coupling of the torsional and bending modes^48^.

For the flexural-torsional flutter treatment of the conveyor belt in our work an assumption is made that, as a result of the bending-torsion oscillations, the restrained belt section has separated from (i.e., lifted off) its supporting rollers.

The lift force F_y_ acting on the restrained section of the belt is determined as

7 $${F}_{y}={C}_{y}^{\varphi }\varphi cq$$

where:

C^φ^_y_ – aerodynamic lift force coefficient (determined by virtual wind tunnel experiments for a given airflow angle); φ – angle of twist; c – belt cross-section length; q = $\frac{{\uprho }_{\text{a}}{\text{v}}_{\text{a}}^{2}}{2}$ – aerodynamic load.

As φ equals zero at both ends of the restrained belt section, its variation is assumed sinusoidal:

8 $$\varphi ={\varphi }_{max}\mathit{sin}\frac{\pi {x}_{(z)}}{l}$$

where l is the length of the restrained section, x_(z)_ is a coordinate along z-axis, and φ_max_ is a maximal value of φ.

Similar to the divergence phenomenon discussed above, for the belt to remain structurally intact in flatter the induced aerodynamic twist must be balanced out by the restoring elastic forces of the material of the belt. Considering Eqs. (7) and (8), the aerodynamic moment M_a_ can now be written out as

9 $${M}_{a}={F}_{y}d={C}_{y}^{\varphi }\varphi cqd={C}_{y}^{\varphi }{\varphi }_{max}cqd\mathit{sin}\frac{\pi {x}_{(l)}}{l}$$

With φ varying sinusoidally, φ_max_ is reached at the mid point of the restrained section (i.e., x_(z)_ = ½ l). From the strength of materials considerations φ can then be determined as

10 $$\varphi ={\int }_{0}^{\raisebox{1ex}{$l$}\!\left/ \!\raisebox{-1ex}{$2$}\right.}\frac{{M}_{a}}{G J}{\text{d}}{\text{z}}$$

where:

G – torsion modulus (i.e. transverse shear modulus, ref. Table 1) of the material of the belt^12^, J – moment of inertia of belt cross-section in torsion.

Recognising that φ_max_ corresponds to the critical speed of flutter (i.e., the airflow speed), substituting M_a_ from Eq. (9) into Eq. (10) and simplifying Eq. (10) we have

11 $$\frac{{C}_{y}^{\varphi }{\varphi }_{max}cqd}{G J}\frac{{l}^{2}}{\pi }={\varphi }_{max}$$

Solving Eq. (11) derives the critical airflow speed of flutter v_a_ = 8.8 km/h, which is similar to the 8.5±2.5 km/h value obtained in the belt divergence analysis. Similar to the divergence approach results, the 8.8 m/s value of the airflow is unrealistic to cause structural belt damage as in their normal service the belts are operated in significantly stronger winds without compromising their integrity. Therefore, the use of the dynamic aeroelasticity (i.e., flutter) treatment of the belt for the analysis of its structural integrity in natural wind events is also deemed unsuitable.

#### Critical speed of separation

Further analysis of the divergence and flutter treatments leads to conclude that their effects of consequence to the belt’s structural integrity can occur only at wind speeds exceeding a certain critical speed of belt’s separation from its supporting rollers, V_sep_ > V_sep.cr_, when the conditions of its wind loading significantly change. Therefore, the speed of separation of the conveyor belt from its supporting rollers V_sep_ becomes the criterion for the probability of these aerodynamic phenomena potentially damaging the restrained belt section.

Considering the inclined part of the belt cross-section closest to the flow as an isolated plate, the condition for the equilibrium of the aerodynamic lift force and the force of weight of the belt is then written as:

12 $$C_{y} \frac{{\rho_{a} V_{sep}^{2} }}{2}A = 3\delta \rho gA$$

with the left and the right sides of the equation accounting for the lift force and the weight of the whole belt section, respectively.

Here:

C_y_ – coefficient of aerodynamic lift; δ – conveyor belt thickness; ρ_a_, ρ – air density and conveyor belt material density; A = c×l – characteristic aerodynamic area of the restrained section of the belt; g – gravitational acceleration.

From Eq. (12), V_sep_ is then obtained as

13 $$V_{sep} = \sqrt {\frac{6\delta \rho g}{{C_{y} \rho_{a} }}}$$

Solving Eq. (13) showed that the separation of the belt from its supporting rollers occurs at a wind speed v_a_ greater than V_sep.cr_ = 37.9 m/s at the α = 0^0^ airflow angle and greater than V_sep.cr_ = 61.6 m/s at α = 30^0^.

The performance of the restrained section of the belt after a v_a_ > V_sep.cr_ wind gust is determined by the rate of v_a_ decay to a safe, i.e., v_a_ $\le$ V_sep.cr_ level and the deflection of the belt after its separation from the supporting rollers. To examine the effects of the airflow on the deformable cross-section of the belt and the effect of belt’s profile geometry change on the airflow field (ref. Sect. “Numerical modeling”), the behavior of a l_cr_ = 16.7 m long restrained section of the belt (i.e., the shortest safest restrained section length value found in Sect. “Numerical modeling”) was modelled in the 37.9 m/s < v_a_ $\le$ 75 m/s airflow range. The two-way fluid–structure interaction (FSI) approach^49^ was used. The double conjugation method using the direct connection between the Lagrangian grid describing the elastic model of the belt^50^ and the Euler grid^35^ for calculating the motion of the airflow used as the basis of this approach. For the conjugation of solutions generated by both software complexes, an explicit splitting method was used, with the calculation process divided into a finite number of steps.

**Fig. 9:**
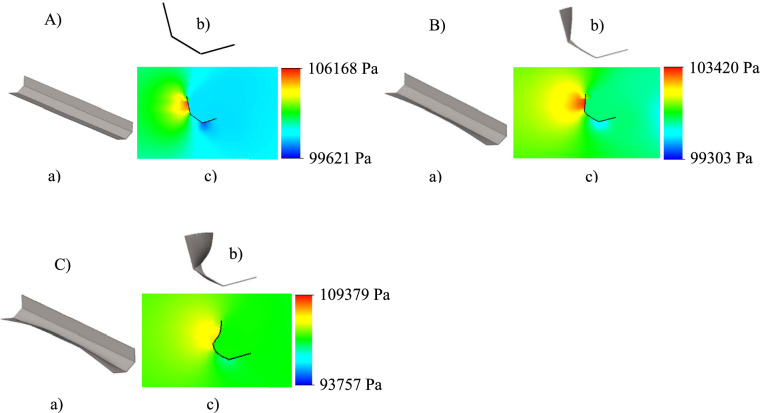
Modeling results for **l**_**cr**_ = 16.7 m at **v**_**a**_ = 75 m/s and **α** = 30^0^ (Note: Airflow transverse to the restrained section, direction left to right). (**A**) first modeling step (i.e., initial undeformed state); (**B**) intermediate step; (**C**) final step (i.e., finally deformed belt); (**a**) restrained section full 3D view; (**b**) mid-length cross-section shape; (**c**) mid-length cross-2D profile pressure distribution.

The FE program complex simulated the kinematics and deformation of the belt during each such time step under the effect of the load obtained from the CFD solver. The displacement of the FE grid nodes that came to the CFD solver from the CAE solver at each time step resulted in a change in the flow region (finite-volume grid) and the calculation of new aerodynamic flow characteristics, including pressure distribution. The advantage of this approach is a completely conservative transfer of physical quantities from one grid to another and a minimal approximation error. At the same time, the convergent process of deformation of the belt to its final (limiting) shape ensured the refined characteristics of the stress-strain state of the belt and the absence of the phenomenon of its divergence occurring in the divergent process of its deformation. Graphic visualization of the results for the restrained belt section is illustrated in Fig. 9.

Referring to Fig. 9, the effect of the aerodynamic load on the restrained section of the belt (Fig. 9A) is counteracted by the restoring (i.e., reactive) forces generated in the elastically deforming belt (Fig. 9B). These restoring forces change the belt’s profile geometry so that a larger area of the belt gets exposed to the airflow. This increase of the belt area exposed to the airflow is accompanied by a proportional reduction in the wind load pressure on the belt and thus results in the aerodynamic load decreasing to safe levels as illustrated by the pressure profile in (Fig. 9C).

## Final remarks and conclusions

In this work, we examined the engineering basis of design of the minimal safe value of the restraint interval to ensure open-air conveyor belt structural integrity under the loading conditions of natural hazardous wind events using all available treatments, including strength of materials and numerical methods and conventional aerodynamic techniques. Each of these treatments has its requisite assumptions and its own merits.

The SM approach required the adoption of several assumptions, including for belt material properties, geometric parameters of the belt, and aerodynamic loading schematics. However, in the case of the unidirectionally reinforced steel cord rubber conveyor belt some of these assumptions do not hold. More specifically, one of the fundamental assumptions of the SM thin-walled beam model, chosen in our work as the most fitting standard structural model representing conveyor belt, is the isotropy of the material of the beam. It is clear though that, for the highly anisotropic (i.e., orthotropic) material of the steel-cord rubber conveyor belt, with some of its directional mechanical properties differing by up to a factor of 10^4^^12^, this assumption is unjustified. Similarly, another requisite assumption of the SM beam model is the non-deformability of the cross-section of the beam. However, in actuality the geometry of the cross-section of the belt between the restraints changes dramatically under the cyclonic wind load. Consequently, and as can be expected, structural integrity analysis of the belt using the SM method with the thin-walled beam model yielded only highly approximate results, with the minimal safe distance between belt restraints ranging between 33.8 m and 17.6 m and with no safety margin build in. On this basis, the SM approach was considered inadequate for the accurate belt restraints interval determination. The results of the SM approach were marginally improved with computer-based NM simulations of deformations of the belt, with the minimal safe distance between belt restraints found to be 17.9 m.

Similarly to the SM approach, both the static (i.e. divergence) and the dynamic (i.e. flutter) aeroelasticity treatments of the belt also required the assumption of the non-deformability of the belt’s profile. It is then understandable that the use of these techniques for the analysis of critical, i.e. causing structural belt damage, airflow velocities, derived similarly unrealistic values. The critical velocity values derived with these methods were approx. 9 m/s which is severalfold lower than wind velocities experienced by the belts in their normal-duty service on industrial sites. Consequently, the use of both the static and the dynamic aeroelasticity treatments for the analysis of the structural integrity of the belt were also deemed unsuitable.

In contrast with the SM, NM, and both conventional aeroelasticity approaches, the most accurate results were obtained from introducing dynamic component of the conveyor belt’s elasticity into the aerodynamic flexural-torsional flutter treatment of the belt, delivering the minimal length of the restrained section of the belt value of 16.7 m at the design safety factor of 1.5. We note that this value is over 36% larger than the 12.2 m current best practice industry standard restraint interval and that adopting this larger value of the interval would generate significant efficiencies for the mining and minerals transportation industry in both capital expenditure and operational costs while at the same time significantly improving personnel safety. Using a hypothetical example of a 5km long open-air overland conveyor, the current industry-standard 12.2m tie-down interval results in 410 restraint points. Assuming that the cost of each restraint point combines the cost of the restraint assembly itself and its engagement in preparation for cyclone and then disengagement post-cyclone by two personnel crew, the total cost per manual restraint unit would average ~$350–$400 AUD. Increasing the tie-down interval from the current 12.2 m to 16.7 m reduces the number of restraints to 299 (i.e. 27% reduction). This, in turn, translates to an estimated cost saving of $39,000–$44,000 AUD for the 5 km long conveyor in focus (i.e. $8,200 AUD per kilometre of the conveyor) per cyclone event. In addition to these direct cost savings and equally importantly, reduced number of belt restraints improves work health and safety environment. This includes reduction of the total exposure time of site personnel to the elements (i.e. over 45°C ambient temperatures and open-sun environment) and significantly lowers the likelihood of injuries due to fatigue, trips and falls on an uneven ground, and muscle strains from manual handling of restraints and their tie-down chains.

The results of our work may also be of relevance to the design and implementation of automated belt restraint systems, which are increasingly considered as an alternative to the manual practices for modernized conveyor infrastructure including both retrofitting the existing systems and design of new conveyors. We envisage that the uptake of the results of our research can thus help further optimize efficiency of open-air belts restraining practices. However, for completeness of consideration of the manual versus automated restraint system merits it should be noted that during the manual restraining and especially freeing-up of conveyor belts by the personnel crews, the crews also carry out visual inspection of the restraints, including their repairs or replacement as necessary. Switching to the automated restraining, while dramatically improving site health and safety environment by removing human personnel from the inherently hazardous task of manual belt restraining, will necessitate development and implementation of a separate asset condition monitoring practice and repairs/replacement service. Given the higher cost and complexity of automated systems compared to traditional manual restraints, this additional consideration will need to be included in the cost-benefit analysis of selecting each site-specific belt restraining system.

We acknowledge that the present study relies exclusively on numerical simulations and does not include physical validation through wind tunnel experiments or field testing. This limitation is primarily due to the specific geometric scale of open-air overland conveyors, with their more than fivefold ratio of the length of the restrained section of the belt to belt width and fiftyfold ratio of belt width to its thickness. This expansive geometric scale presents substantial challenges in achieving dynamic similarity and replicating boundary conditions in physical experiments in a laboratory setting. Moreover, conducting controlled experiments on mining infrastructure in field testing during actual cyclonic events is also highly problematic due to restricted site access for obvious health and safety considerations and the inherent inability to control or reproduce atmospheric conditions. Nevertheless, we emphasize that the developed numerical methodology offered in our paper may serve as a foundation for future physical validation efforts, including testing in custom-designed wind tunnel setups or field trials, subject to the appropriate technical and financial resources. Incorporating such experimental data will help strengthen the predictive capabilities of the proposed approach and further refine the optimal restraining interval for open-air conveyor belts exposed to natural hazardous wind events.

While the aerodynamic modeling in this study was based on the assumption of laminar airflow around the restrained belt section, we recognize that turbulent, unsteady airflow is typical under cyclone conditions. This simplification was made intentionally to establish a baseline and enable tractable analysis across various airflow angles and speeds. However, we acknowledge that omitting turbulence may lead to overestimation of structural stability due to the absence of gust-induced fluctuations in the loading. In future studies, the inclusion of turbulence modelling – such as by means of Reynolds-Averaged Navier-Stokes (RANS) or Large Eddy Simulation (LES) – is planned to refine the pressure field representation and improve predictive accuracy of belt deformation and failure risk under real cyclonic wind patterns. This step is essential to enhance the model’s fidelity and practical applicability.

Based on our work, we propose that the above approach of incorporating dynamic component of the belt’s elasticity into the aerodynamic flexural-torsional flutter treatment of the belt should be used at the design stage of overland conveyor systems. This will provide the basis for better management of the belt restraining practices and ensure structural integrity of the belts under the conditions of natural hazardous wind events.

Overall, the results of our work in the validation of suitability of the available engineering approaches for examination of structural integrity of the reinforced rubber conveyor belts improve the understanding of the behavior of the belts under the irregular aerodynamic loading conditions. Finally, we note that, to the best of our knowledge the work presented here is the first ever attempt at a thorough analysis of aerodynamic behavior of overland conveyor belt, i.e., a critical infrastructure asset for the Australian and global mining and minerals transportation industry, under the loading conditions of the natural hazardous wind events such as tornados and tropical cyclones.

## Data Availability

The authors declare that the data supporting the findings of this study are available within the paper.
